# Frequency and Prognostic Impact of Local Ablation Therapy for Oligoprogression in Non‐Small Cell Lung Cancer

**DOI:** 10.1111/1759-7714.70119

**Published:** 2025-07-08

**Authors:** Daisuke Morinaga, Jun Sakakibara‐Konishi, Ryohei Kamada, Masahiro Kashima, Kosuke Tsuji, Shotaro Ito, Megumi Furuta, Tetsuaki Shoji, Yuta Takashima, Hidenori Kitai, Yasuyuki Ikezawa, Hiroshi Taguchi, Tatsuya Kato, Yoshiki Shinomiya, Kanako C Hatanaka, Yutaka Hatanaka, Satoshi Konno

**Affiliations:** ^1^ Department of Respiratory Medicine Faculty of Medicine and Graduate School of Medicine, Hokkaido University Sapporo Japan; ^2^ Medical Network and Welfare Center Hokkaido University Hospital Sapporo Japan; ^3^ Department of Radiation Oncology Hokkaido University Hospital Sapporo Japan; ^4^ Department of Thoracic Surgery Hokkaido University Hospital Sapporo Japan; ^5^ Center for Development of Advanced Diagnostics Hokkaido University Hospital Sapporo Japan

**Keywords:** genetic analysis, local ablation therapy, non‐small cell lung cancer, oligometastasis, oligoprogression

## Abstract

**Background:**

During the systemic treatment of patients with non‐small cell lung cancer (NSCLC), oligoprogression (OP), a condition in which most lesions remain controlled while a few progress or develop, has recently attracted attention. Traditionally, systemic therapy is continued after disease progression; however, advancements in local ablation therapy (LAT), such as radiotherapy and surgery, have demonstrated clinical efficacy in patients with OP. The characteristics of patients who may benefit from LAT or their genetic background remain unclear. This study evaluated the frequency, clinicopathological characteristics, and efficacy of LAT in the treatment of OP.

**Methods:**

A retrospective review was conducted of 510 patients with NSCLC who experienced disease progression after systemic therapy.

**Results:**

Overall, 106/510 (23.6%) patients exhibited OP; among these, six patients who received only the best supportive care after OP were excluded. Systemic therapy alone was administered to 79 patients (79.0%), while 21 (21.0%) received LAT. Median local progression‐free survival was numerically longer in the LAT group than in the systemic therapy‐only group (8.3 and 6.7 months, respectively; *p* = 0.38). In addition, overall survival was also numerically longer in the LAT group than in the systemic therapy‐only group (78.1 and 55.1 months, respectively; *p* = 0.57). Ribonucleic acid sequencing revealed an increase in extracellular matrix‐related gene expression after OP, providing potential molecular insights.

**Conclusions:**

Although this study found no significant prognostic benefit of LAT in patients with OP, future research integrating clinical and molecular data may identify patients most likely to benefit from LAT.

## Graphical abstract

OP occurred in 23.6% of patients, reaffirming its clinical prevalence. LAT did not significantly prolong OS in OP patients, though trends suggested benefit in LPFS and OP‐OS. Repeat OP occurred in 45%, potentially indicating a more indolent, favorable prognosis subgroup.

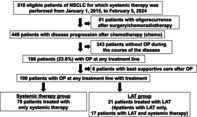

## 1 Introduction

Lung cancer remains the leading cause of cancer‐related deaths worldwide [1, 2]. Non‐small cell lung cancer (NSCLC) accounts for approximately 80% of all lung cancer cases [3]. Although advances in treatment are improving prognosis, the disease remains rarely curable, with a 5‐year survival rate ranging from 20% to 30% [4, 5, 6, 7]. Efforts to improve the prognosis of NSCLC have included exploring local ablation therapies (LAT) as potential adjuncts to systemic treatments [8].

Oligometastasis (OM), a concept proposed by Hellmann et al., refers to a state in which only a limited number of metastatic lesions are present in advanced‐stage disease [9, 10]. Oligoprogression (OP), a subset of OM, describes the occurrence of a small number of progressive lesions during systemic therapy. Previous reports suggest that OP occurs in 20%–40% of cases, with frequencies influenced by factors such as the presence of driver genes and the type of treatment, including tyrosine kinase inhibitors (TKI) or immune checkpoint inhibitors (ICI) [11, 12, 13, 14, 15]. OP lesions are believed to represent populations resistant to systemic therapy. Eliminating these lesions using LAT may prolong survival or extend the duration of systemic therapy. This rationale supports the application of LAT in the management of OP. However, the clinical characteristics of individuals most likely to benefit from LAT remain unclear. Only a few prospective trials have demonstrated the efficacy of LAT in patients with OP in NSCLC [16]. Moreover, retrospective studies comparing outcomes between LAT and non‐LAT groups are scarce [12, 15]. Therefore, validating the utility of LAT in real‐world settings and identifying patient characteristics associated with a greater likelihood of benefit are essential. LAT includes surgery, radiotherapy, and transthoracic lung ablation techniques such as microwave ablation (MWA), radiofrequency ablation (RFA), and cryoablation [13, 15, 17, 18, 19]. Surgery remains the treatment of choice for small‐sized lung cancers, and the efficacy of limited surgical procedures, such as sublobar resection or segmentectomy, in addition to lobectomy, has been demonstrated [20, 21]. Surgery offers the advantage of being curative, while also allowing for pathological and genetic evaluation of the lesion. For patients who are inoperable, such as the elderly or those with poor pulmonary function or performance status, radiotherapy or transbronchial lung ablation is often selected. Radiation therapy is supported by extensive evidence and is widely used for lung cancer, with a high local control rate [22]. Its toxicity is relatively low and typically includes pneumonitis or dyspnea. A phase II trial reported that surgery was selected as part of LAT in 28% of patients (including 24% who received both radiation and surgery), while the remaining patients were treated with radiotherapy [23]. Transbronchial lung ablation is also considered a therapeutic option for early‐stage or recurrent lung cancer. RFA is the most commonly used thermal ablation technique; by rapidly increasing the temperature of tumor tissue, RFA induces tumor cell necrosis and leads to favorable clinical outcomes. MWA operates at higher energy and temperatures than RFA. The complications of RFA and MWA are generally mild and include pneumothorax, pleural effusion, subcutaneous emphysema, and intra‐alveolar hemorrhage [24]. Cryoablation, a cold‐based thermal ablation technique, has similar complications to those of RFA and MWA, though it is generally associated with less pain [25]. Several prospective and retrospective studies have evaluated the efficacy and safety of various transthoracic lung ablation methods in OM [26]. However, direct comparisons of these modalities are challenging due to patient selection bias. Previous reports suggest that OM patients treated surgically may have better prognoses than those treated with radiation therapy, although the sample sizes have been small, and further prospective validation is needed [27]. Additionally, with the advent of ICI as systemic therapy, synergistic effects with LAT, known as the abscopal effect, are particularly anticipated in the context of radiation therapy [15]. However, limited research has explored the interaction between LAT and systemic treatments, and the optimal sequencing of these therapies remains unclear. Consequently, the choice and timing of LAT interventions are often determined by the expertise and resources available at each institution.

Additionally, reports on the genetic characteristics of patients with OM remain limited. Liao et al. found that patients with OM exhibited significantly lower mutation frequencies in genes such as *ERBB2*, *ALK*, *MLL4*, *PIK3CB*, and *TOP2A* compared to those with polymetastatic disease [28]. De et al. found that EGFR mutations were associated with longer overall survival (OS), whereas *STK11* mutations were linked to shorter progression‐free survival (PFS) in patients with OM [29]. However, these studies have primarily focused on synchronous OM. Furthermore, genetic analyses of liver OM have been reported, including a few cases of OP; however, these studies were limited and did not provide a clear characterization of OP cases [30]. Understanding the genetic features of patients who are more likely to progress locally, rather than through systemic dissemination, would be extremely beneficial for tailoring treatments for OP.

This study aimed to determine the frequency of OP and evaluate the therapeutic effects of LAT in patients with OM. Furthermore, we investigated the clinical and molecular characteristics of patients with OP to better understand this distinct clinical entity.

## 2 Materials and Methods

### 2.1 Study Design and Patient Selection

This retrospective observational study was conducted at Hokkaido University Hospital. The inclusion criteria were patients aged 20 years or older who were diagnosed with NSCLC, experienced relapse after systemic therapy, and were diagnosed with OP between January 2015 and February 2024. OP was defined as the presence of three or fewer new or exacerbating lesions [13]. Patients with pleural or pericardial effusion, pleural dissemination, or carcinomatous lymphangitis were classified as non‐OP cases. The key exclusion criteria were individuals who did not receive any treatment following the diagnosis of OP. Additionally, patients with disease recurrence after radical treatments, such as surgery or radiation therapy, and those presenting with a small number of metastases, referred to as oligorecurrence, were excluded due to potential differences in patient backgrounds. LAT was defined as surgery or radiation therapy targeting OP lesions performed for curative or palliative purposes. Microwave coagulation therapy (MCT)/MWA is not reimbursed by Japan's National Health Insurance for the treatment of lung cancer. Although RFA was approved for reimbursement in 2022, implementing RFA at our institution has required considerable time, and as a result, the procedure has rarely been performed. Consequently, no patients in this study underwent transthoracic tumor ablation. A multidisciplinary board confirmed the feasibility of LAT. Patients who underwent LAT targeting only a subset of oligoprogressing lesions were also included in the LAT group for analysis. All participants provided informed consent. The study protocol was approved by the Ethics Review Board of our institution (Approval Number: 023‐0474). This study was conducted in accordance with the principles of the Declaration of Helsinki.

The data cut‐off date was April 30, 2024. The primary endpoint was the proportion of patients with NSCLC who developed OP after systemic therapy. The secondary endpoints included the evaluation of the clinical and genetic characteristics of patients with OP and the prognostic influence of LAT on OP.

### 2.2 RNA Sequencing

Total ribonucleic acid (RNA) was isolated from formalin‐fixed paraffin‐embedded tissue sections of each case using AllPrep DNA/RNA Kits (QIAGEN, the Netherlands) according to the manufacturer's instructions. The samples were confirmed to have a purity of at least OD260/280 1.6 and OD260/230 1.6, with no degradation or contamination confirmed by TapeStation (Agilent Technologies, USA). The extracted total RNA was sent to Takara Bio Inc. (Shiga, Japan) for RNA sequencing (RNA‐seq) analysis. RNA‐seq was performed using an Illumina NovaSeq 6000 instrument (Illumina, USA). Library quality was assessed using FASTQC (version 0.12.1) and fastp (version 0.23.4). Clean reads were pseudo‐aligned, and gene expression levels were quantified using the Kallisto tool (version 0.50.1). Quantified data were loaded into R (version 4.4.2) via the tximport package (version 1.34.0), and differential expression analysis was performed using the DESeq2 package (version 1.46.0). Differentially expressed genes were identified using a log2 fold change > 2.0, or log2 fold change < −2.0, and *p* < 0.05. Gene ontology (GO) analysis was conducted, and the results were visualized using the clusterProfiler package (version 4.14.4).

### 2.3 Statistical Analyses

Categorical data were summarized as frequencies and percentages, whereas continuous data were reported as medians with ranges. The *χ* ^2^ test and Fisher's exact test were used to compare categorical variables, and the Wilcoxon rank‐sum test was used to compare continuous variables. The overall response rate (ORR) was defined as the proportion of patients achieving either a complete or partial response to treatment based on the Response Evaluation Criteria in Solid Tumors version 1.1 [31]. PFS was defined as the time from the diagnosis of OP to PD or the date of last known survival. Local PFS (LPFS) was defined as the time from the diagnosis of OP to PD in the organ where OP was confirmed. OS was defined as the time from the start of first‐line systemic therapy to the date of death or last known survival. OP‐OS was calculated from the date of OP diagnosis to death or the last follow‐up. PFS, LPFS, OS, and OP‐OS were evaluated using the Kaplan–Meier method and compared using the two‐sided log‐rank test. Hazard ratios (HRs) and 95% confidence intervals (CIs) were estimated using the Cox proportional hazard regression model. Variables with *p* < 0.05 in the univariate analysis were further examined in the multivariate analysis. All statistical tests were two‐sided, and statistical significance was set at *p* < 0.05. All statistical analyses were performed using JMP Pro 17.0 software (SAS Institute Inc., USA).

## 3 Results

### 3.1 Patient Characteristics

**FIGURE 1:**
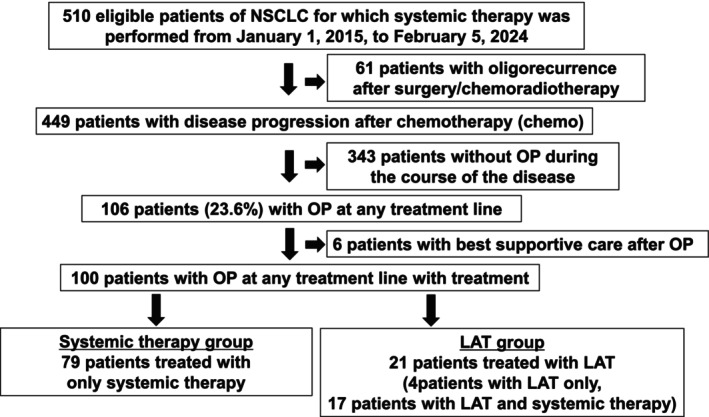
Patient flowchart. LAT, local ablation therapy; NSCLC, non‐small cell lung cancer; OP, oligoprogressive.

We screened 510 patients with NSCLC who received systemic therapy and excluded 61 who experienced oligorecurrence after radical treatment (Figure 1). Among them, 106 (23.6%) experienced OP after systemic therapy. Six patients were further excluded as they received only palliative therapy after OP, leaving 100 patients included in the analysis. The cohort was divided into two groups: 79 patients who received only systemic therapy when OP occurred (systemic therapy group) and 21 patients who underwent additional LAT (LAT group).

Patient characteristics are summarized in Table 1. No significant differences were observed in patient characteristics between the two groups. PD‐L1 expression and driver mutation status were assessed at the time of lung cancer diagnosis. Details of these driver mutations and other gene alterations in the patients examined using next‐generation sequencing are shown in Table S1. EGFR mutations were the most common in both groups, with no significant difference in frequency (Table 1). In Japan, after ICIs became available in clinical practice in 2016, there was a period during which ICIs could be administered after second‐line treatment regardless of PD‐L1 expression. Additionally, among the patients evaluated in this study, approximately half were positive for driver mutations, and these patients often did not receive ICIs as first‐line therapy. As a result, PD‐L1 expression was not examined in most cases with driver mutations. PD‐L1 expression was not significantly different between the two groups. Most of LAT was radiation therapy (15 out of 21 patients) and there was one patient that underwent consolidative radiation therapy after surgery. The lung is the most frequent organ when disease progression occurs in a single organ. Treatment choices differed depending on the organ involved, with systemic therapy being more frequent in patients with lung lesions, whereas LAT was significantly more common in patients with brain metastases and numerically higher in those with bone metastases (Table 2).

**TABLE 1:** Characteristics of patients with oligoprogression treated with or without local ablation therapy.

	Overall (*n* = 100)	Systemic treatment group (*n* = 79)	LAT group (*n* = 21)	*p*
Patients' age at OP diagnosis (median)	70 (41–84)	70 (41–81)	68 (41–84)	0.93
Sex				0.62
Male	55 (55%)	42 (53.2%)	13 (61.9%)	
Female	45 (45%)	37 (46.8%)	8 (38.1%)	
Smoking history				1.0
Ever	73 (73%)	58 (73.4%)	15 (71.4%)	
Never	27 (27%)	21 (26.6%)	6 (28.6%)	
Prior radical surgery for lung cancer				1.0
Yes	12 (12%)	10 (12.7%)	2 (9.5%)	
No	88 (88%)	69 (87.3%)	19 (90.5%)	
Prior chemoradiotherapy				0.58
Yes	5 (5.0%)	5 (6.3%)	0 (0%)	
No	95 (95%)	74 (93.7%)	21 (100%)	
Clinical stage at diagnosis				0.87
I	5 (5.0%)	4 (5.1%)	1 (4.8%)	
II	5 (5.0%)	4 (5.1%)	1 (4.8%)	
III	26 (26%)	22 (27.8%)	4 (19.0%)	
IV	64 (64%)	49 (62%)	15 (71.4%)	
Treatment line at OP				0.79
First line	73 (73%)	57 (72.2%)	16 (76.2%)	
Second and subsequent line	27 (27%)	22 (27.8%)	5 (23.8%)	
Median PFS of previous therapy (months) (95% CI)				0.26
	9.3 (6.3–9.3)	7.6 (6.3–10.3)	6.6 (5.1–8.2)	
Histology				0.97
Adeno	72 (72%)	56 (70.9%)	16 (76.2%)	
Squamous	24 (24%)	19 (24.1%)	5 (23.8%)	
NOS	2 (2.0%)	2 (2.5%)	0 (0%)	
Others	2 (2.0%)	2 (2.5%)	0 (0%)	
Driver mutations				0.46
With	43 (43%)	32 (40.5%)	11 (52.4%)	
Without	57 (57%)	47 (59.5%)	10 (47.6%)	
EGFR sensitizing mutations				0.60
With	32 (32%)	24 (30.4%)	8 (38.1%)	
Without	68 (68%)	55 (69.6%)	13 (61.9%)	
PD‐L1 expression (22C3)				0.40
< 1%	15 (15%)	13 (16.5%)	2 (9.5%)	
1%–49%	11 (11%)	10 (12.7%)	1 (4.8%)	
≥ 50%	17 (17%)	14 (17.7%)	3 (14.3%)	
Unknown	57 (57%)	42 (53.1%)	15 (71.4%)	
Modality of LAT				
Radiation			15 (71.4%)	
Surgery			5 (23.8%)	
Radiation + surgery			1 (4.8%)	

Abbreviations: EGFR, epidermal growth factor receptor; LAT, local ablation therapy; NOS, not otherwise specified; OP, oligoprogression.

**TABLE 2:** Details of progressing/newly appearing lesions at oligoprogression.

Characteristics	No (%)			
	All	Systemic treatment group	LAT group	*p*
	*n* = 100	*n* = 79	*n* = 21	
Number of progressing/newly appearing lesions				0.15
1 lesion	67 (67%)	50 (63.3)	17 (81.0)	
2 lesions	26 (26%)	24 (30.4)	2 (9.5)	
3 lesions	7 (7%)	5 (6.3)	2 (9.5)	
Organ type when disease progressed in a single organ				
Lung	68 (68.0%)	62 (78.5%)	6 (28.6%)	< 0.01
Liver	2 (2.0%)	2 (2.5%)	0 (0%)	1.00
Brain	9 (9.0%)	1 (1.3%)	8 (38.1%)	< 0.01
Adrenal	2 (2.0%)	2 (2.5%)	0 (0%)	1.00
Bone	5 (5.0%)	2 (2.5%)	3 (14.3%)	0.06

Abbreviation: LAT, local ablation therapy.

### 3.2 Prognostic Impact of OP

**FIGURE 2:**
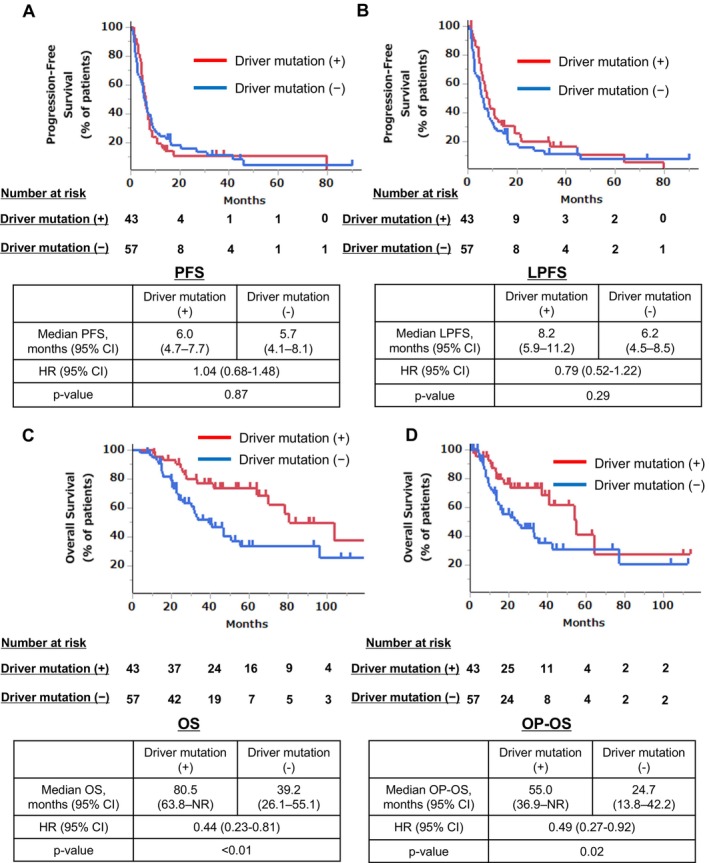
Kaplan–Meier curves and estimated median progression‐free survival (A), local progression‐free survival (B), overall survival (C), and overall survival after oligoprogression (D) in the presence or absence of driver mutations. Chemo, chemotherapy; CI, confidence interval; HR, hazard ratio; LAT, local ablation therapy; LPFS, local progression‐free survival; OP, oligoprogression; OP‐OS, overall survival after oligoprogression; OS, overall survival; PFS, progression‐free survival.

We examined the prognosis of patients with and without driver mutations. The PFS did not significantly differ between the two groups (HR = 1.04, 95% CI: 0.68–1.48), with a median PFS of 6.0 months in the driver mutation‐positive group and 5.7 months in the driver mutation‐negative group (*p* = 0.87) (Figure 2A). The median LPFS also showed no significant difference between both groups (HR = 0.79, 95% CI: 0.52–1.22), with a median LPFS of 8.2 months in the driver mutation‐positive group and 6.2 months in the driver mutation‐negative group (*p* = 0.29) (Figure 2B). The median OS was significantly longer in the driver mutation‐positive group (HR = 0.44, 95% CI: 0.23–0.81), with a median OS of 80.5 months compared to 39.2 months in the driver mutation‐negative group (*p* < 0.01) (Figure 2C). Similarly, the median OP‐OS was significantly longer in the driver mutation‐positive group (HR = 0.49, 95% CI: 0.27–0.92), with a median OP‐OS of 55.0 months compared to 24.7 months in the driver mutation‐negative group (*p* = 0.02) (Figure 2D).

### 3.3 Prognostic Impact of LAT in Patients With OP

**FIGURE 3:**
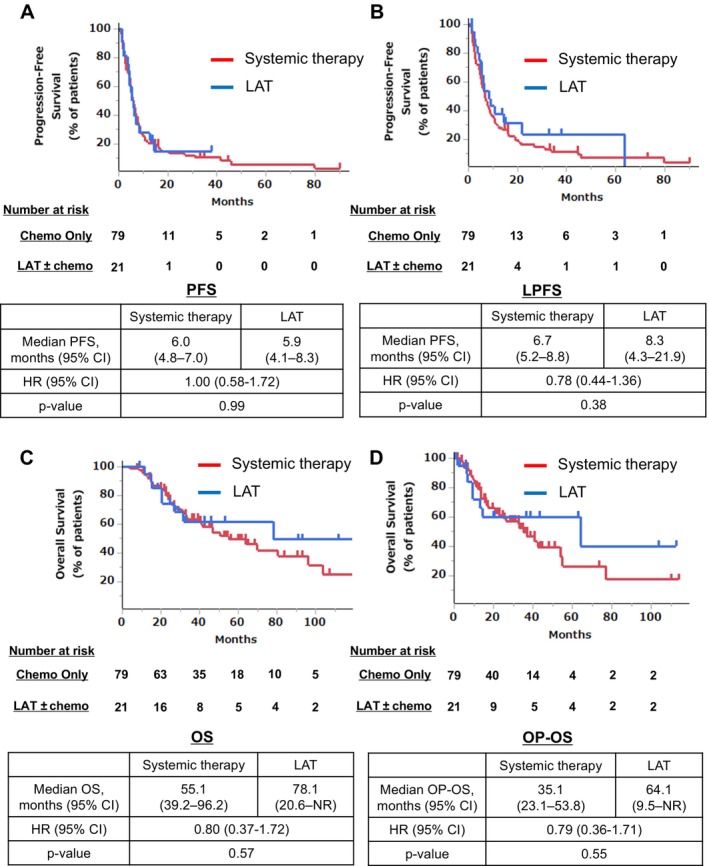
Kaplan–Meier curves and estimated median progression‐free survival (A), local progression‐free survival (B), overall survival (C), and overall survival after oligoprogression (D) by treatment modality. CI, confidence interval; HR, hazard ratio; LAT, local ablation therapy; LPFS, local progression‐free survival; OP, oligoprogression; OP‐OS, overall survival after oligoprogression; OS, overall survival; PFS, progression‐free survival.

We examined the prognostic impact of LAT in patients with OP by comparing the LAT and systemic therapy groups. The prognosis for each group was as follows: the median PFS did not significantly differ between the groups (HR = 1.00, 95% CI: 0.58–1.72), with a median PFS of 6.0 months in the systemic therapy group and 5.9 months in the LAT group (*p* = 0.99) (Figure 3A). The median LPFS was numerically longer in the LAT group without a significant difference (HR = 0.78, 95% CI: 0.44–1.36), with a median LPFS of 6.7 months in the systemic therapy group and 8.3 months in the LAT group (*p* = 0.38) (Figure 3B). The median OS did not show a significant difference (HR = 0.80, 95% CI: 0.37–1.72), with a median OS of 55.1 months in the systemic therapy group and 78.1 months in the LAT group (*p* = 0.57) (Figure 3C). The median OP‐OS was numerically longer in the LAT group (HR = 0.79, 95% CI: 0.36–1.71), with a median OP‐OS of 35.1 months in the systemic therapy group and 64.1 months in the LAT group (*p* = 0.55), although the difference was not significant (Figure 3D). We examined the prognostic impact of LAT separately in patients with and without driver mutations. No significant survival improvement was observed with the addition of LAT in both patients with (Figure S1A–D) and without driver mutations (Figure S2A–D).

### 3.4 Treatment Outcome in Patients With OP

The treatment outcomes in the systemic therapy and LAT groups are shown in Table 3. The ORRs were 23.8% and 34.1% in the systemic therapy and LAT groups, respectively, with no significant difference (*p* = 0.44). Disease control rates were 73.9% and 85.3% in the systemic therapy and LAT groups, respectively (*p* = 0.77). We also examined the patterns of disease progression after treatment for OP. The frequency of patients with progression of pre‐existing disease was 17.7% in the systemic therapy group and 0% in the LAT group.

**TABLE 3:** Treatment outcomes in patients with oligoprogression.

	Overall (*n* = 100)	Systemic therapy group (*n* = 79)	LAT group (*n* = 21)	*p*
Overall response rate (%)	32.0	23.8	34.1	0.44
Disease control rate (%)	77.0	76.0	81.0	0.77
Type of progression				0.01
Organs with pre‐existed lesions (%)	14.0	17.7	0	

Abbreviation: LAT, local ablation therapy.

### 3.5 Frequency and Prognostic Impact of Repeat OP

We defined repeat OP as patients with recurrence of OP after treatment for OP and examined its frequency. Of all the progression events, 36 (45.0%) patients had repeat OP. The incidence of repeat OP was seven cases (40.9%) in the systemic therapy group and 29 (46.7%) in the LAT group (*p* = 0.78), with no significant difference between the groups (Figure S3). When comparing the OP‐OS between the repeat OP group and the nonrepeat OP group, a numerically better trend was observed in the repeat OP group (HR 0.55, 95% CI: 0.29–1.04), with a median OP‐OS of 64.1 months for the repeat OP group and 24.5 months for the non‐repeat OP group (*p* = 0.06) (Figure S4).

### 3.6 Prognostic Factors of Patients With OP

We performed univariate and multivariate analyses to identify the factors associated with OP‐OS (Table S2). In univariate analysis, factors such as the absence of driver mutations, OP limited to liver lesions, and PFS of previous treatment < 6 months were significantly associated with worse OP‐OS. In multivariate analysis, the absence of driver mutations and PFS of previous treatment < 6 months were significantly correlated with worse OP‐OS.

### 3.7 Genetic Expression Profiles of Patients With OP

**FIGURE 4:**
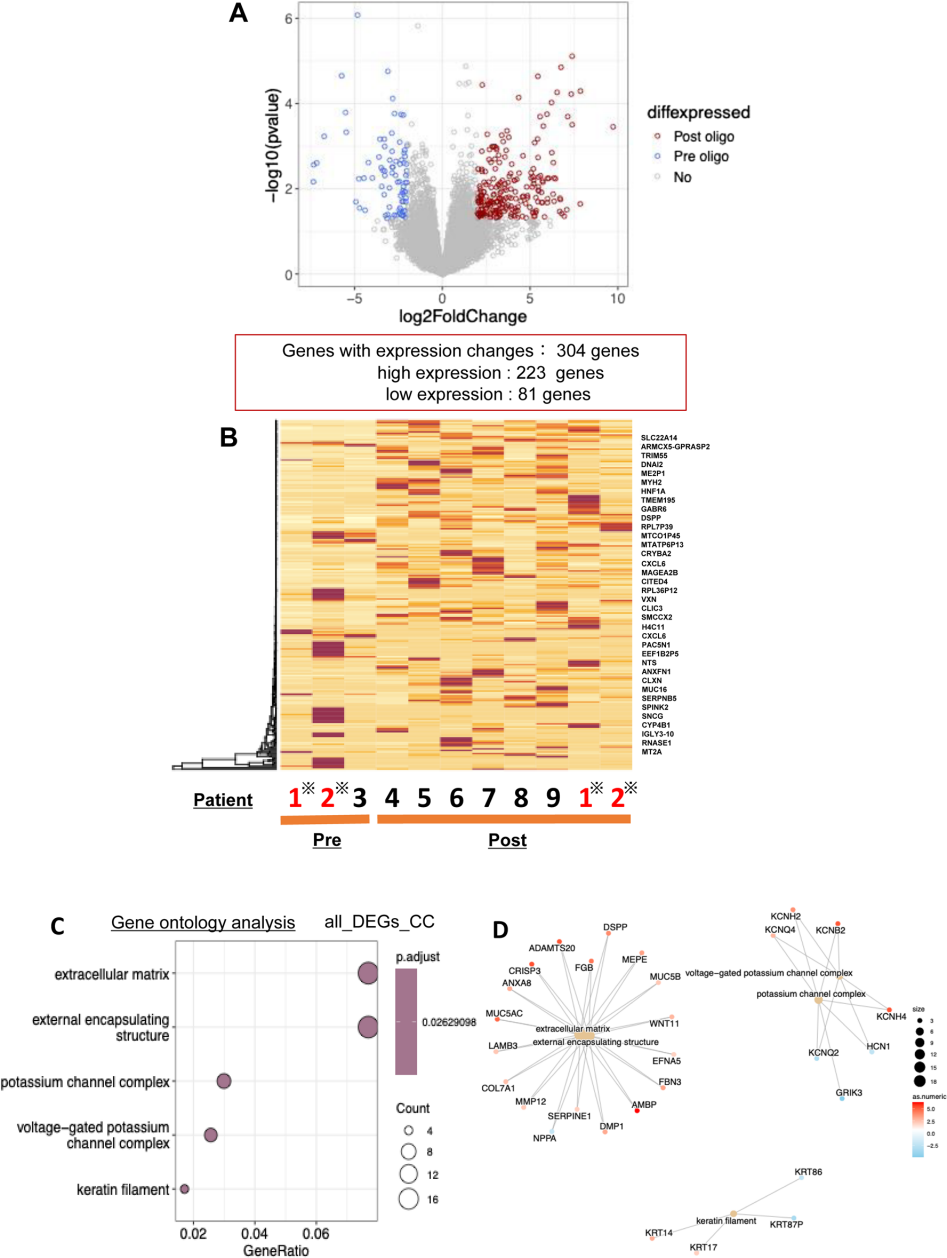
Volcano plot of genes that changed in expression before and after oligoprogression (A). Heat map of gene expression in each patient before and after oligoprogression (B). Patients with asterisks (*) are those for whom samples were obtained at diagnosis and after oligoprogression. Gene ontology (GO) analysis of patients before and after oligoprogression (C). Cnetplot of GO analysis results (D). This plot shows the GO terms extracted based on the genes whose expression changed significantly before and after oligoprogression, as well as the genes included in these terms and their fold changes.

We performed RNA‐seq to explore the genetic expression profiles in patients with OP using specimens obtained both at diagnosis and after OP, which were of sufficient quality and quantity. The characteristics of the patients who underwent RNA‐seq are shown in Table S3. A volcano plot (Figure 4A) comparing gene expression at diagnosis and after OP identified 223 overexpressed genes and 81 under‐expressed genes in post‐OP tissues. Heatmaps (Figure 4B) showing gene expression at diagnosis and after OP for each patient did not reveal any consistent trends. GO analysis (Figure 4C) showed an increase in gene expression related to the extracellular matrix (ECM), external encapsulating structures, and keratin filaments in post‐OP specimens compared to those at diagnosis (Figure 4D).

## 4 Discussion

In this study, more than 20% of patients exhibited OP. Median LPFS was numerically longer in the LAT group compared to the systemic therapy‐only group. Similarly, OS was also numerically longer in the LAT group. The frequency of OP after systemic therapy was 23.6%, which was slightly lower than that reported in previous reports [11, 12, 13, 14]. This discrepancy may stem from the definition of OP used in this study, which adhered to the criteria for three or fewer lesions. In contrast, many other reports have included definitions allowing for up to five lesions. Currently, there is no standardized definition of OP. In recent years, the European Society for Radiotherapy and Oncology has advocated the definition of OM [32]. However, due to their complexity and lack of validated prognostic correlation, a simplified definition was recently proposed [33]. This study adopted the definition of three or fewer to ensure feasibility in performing LAT, potentially identifying a subgroup of patients better suited for LAT. Other factors affecting OP frequency include the presence of driver mutations and treatment type. One report found a 77% frequency of OP after first‐line osimertinib treatment in patients with EGFR mutations [34]. Targeted therapies often achieve high response rates and long‐term disease control, potentially increasing the likelihood of OP, although no consensus has been reached. In this study, no restrictions were imposed on driver mutations or treatment types, resulting in a heterogeneous cohort. Approximately half of the patients tested positive for driver mutations, and the treatment regimens differed, including TKI and ICI therapies. Despite this variability, our findings reaffirm that approximately ≥ 20% of patients with NSCLC may develop OP during treatment in clinical practice.

Our results did not identify a group of patients who showed significant OS prolongation with the addition of LAT. This outcome aligns with several retrospective studies and a systematic review indicating that OP does not benefit from LAT compared with other subtypes of OM [33, 35, 36]. In contrast, a study evaluating the efficacy of LAT in patients with OP demonstrated a significant PFS extension in a subgroup analysis of patients with NSCLC [16]. Discrepancies among studies may result from various factors, such as heterogeneity of patient populations, differences in OP definitions, variations in LAT modalities, and publication bias. Moreover, although not statistically significant, we observed a trend toward prolonged LPFS and OP‐OS in patients who underwent LAT. Although most retrospective studies have analyzed PFS and OS, LPFS and OP‐OS are expected to reflect the benefits of LAT more accurately. In this study, some patients in the LAT group achieved long‐term survival. This trend may be attributable to the ability of LAT to effectively suppress disease progression in treated organs, as observed in this study.

When comparing patient backgrounds between the systemic therapy and LAT groups, brain and bone metastases were more frequent in the LAT group, whereas lung metastases were significantly more frequent in the systemic therapy group. This difference may reflect the broader application of LAT in clinical practice for brain and bone metastases, as it is used for both curative and palliative purposes. For patients with brain metastases, many reports have shown that the addition of LAT to brain metastases can improve prognosis [37, 38]. However, several uncertainties remain regarding pulmonary OP, including whether LAT provides any benefit and whether radiation or surgery demonstrates greater effectiveness for treatment. Further studies are required to determine the potential utility of LAT in pulmonary lesions.

Repeat OP is classified as a variant of OM [32]. Previous studies reported that 26.9%–72.7% of cases involved repeat OP [11, 12, 13]. In our study, 45.0% of patients with OP experienced repeat OP, indicating that repeat OP is a relatively common occurrence. However, reports on the prognostic effects of repeat OP are limited. A previous report comparing the prognosis of repeat OP with and without LAT found significantly improved PFS and OS in patients treated with LAT [11]. Although our study included patients who underwent LAT and those who did not within the repeat OP group, their prognosis appeared to be numerically better than that of the non‐repeat OP group. Based on our findings and the aforementioned report, repeat OP may exhibit an indolent nature and be associated with a better prognosis.

We examined gene expression characteristics in patients with OP. GO analysis showed increased expression of genes related to the ECM and keratin filaments in patients after OP. The ECM is involved in the promotion of metastasis and progression of various carcinomas by interacting with tumor, stromal, and immune cells [39, 40, 41]. However, the relationship between ECM and OM or OP remains largely unexplored and warrants further investigation. OP may have genetically distinct characteristics, especially with respect to genes encoding metastasis and disease progression. Elucidating these genetic characteristics may aid in selecting appropriate patients with OP to consider the treatment modality.

This study had several limitations. First, this was a single‐center, retrospective study involving a small number of patients. In particular, RNA‐seq analysis relied on a limited number of cases, which may have been greatly influenced by individual variability. Second, LATs conducted in this study aimed to provide palliative effects rather than achieving a cure, requiring caution in interpreting the outcomes of LAT for OP. Given the small number of patients in the LAT group, it is difficult to conclude that the efficacy of LAT can be adequately validated. In addition, radiotherapy was the predominant LAT modality in this study, making it impossible to compare its efficacy and safety with that of surgery. Finally, the definition of OP was not well established, and the applied criterion of three or fewer lesions remained unverified for evaluating LAT.

## 5 Conclusions

The addition of LAT to OP in patients with NSCLC had a minimal impact on prognosis, although a trend toward improved local control and prolonged OS was observed. Additionally, repeat OP may represent a subgroup associated with favorable prognoses.

## Author Contributions

**Daisuke Morinaga:** conceptualization, methodology, investigation, data curation, resources, writing – original draft. **Jun Sakakibara‐Konishi:** conceptualization, methodology, data curation, resources, writing – review and editing, supervision. **Ryohei Kamada:** investigation, writing – review and editing. **Masahiro Kashima:** investigation, writing – review and editing. **Kosuke Tsuji:** investigation, writing – review and editing. **Shotaro Ito:** investigation, writing – review and editing. **Megumi Furuta:** investigation, writing – review and editing. **Tetsuaki Shoji:** investigation, writing – review and editing. All authors had full access to the data in the study and take responsibility for the integrity of the data and the accuracy of the data analysis.

## Ethics Statement

The study protocol was approved by the Ethical Review Board for Life Science and Medical Research of Hokkaido University Hospital (approval number: 023‐0474, dated September 22, 2024). This study was conducted in accordance with the tenets of the Declaration of Helsinki. The data cut‐off date was April 30, 2024. Consent was obtained for the use of specimens.

## Conflicts of Interest

The authors declare no conflicts of interest.

## Supporting information


**Figure S1.** Kaplan–Meier curve and estimated median progression‐free survival (A), local progression‐free survival (B), overall survival (C), and overall survival after oligoprogression (D) by treatment modality in patients with driver mutations.
CI, confidence interval; HR, hazard ratio; LAT, local ablation therapy; LPFS, local progression‐free survival; OP, oligoprogression; OP‐OS, overall survival after oligoprogression; OS, overall survival; PFS, progression‐free survival.

**Figure S2.** Kaplan–Meier curve and estimated median progression‐free survival (A), local progression‐free survival (B), overall survival (C), and overall survival after oligoprogression (D) by treatment modality in patients without driver mutations.
CI, confidence interval; HR, hazard ratio; LAT, local ablation therapy; LPFS, local progression‐free survival; OP, oligoprogression; OP‐OS, overall survival after oligoprogression; OS, overall survival; PFS, progression‐free survival.

**Figure S3.** Patient flowchart after treatment for oligoprogression.
OP, oligoprogression.

**Figure S4.** Kaplan–Meier curve and estimated median overall survival after oligoprogression of patients with repeat oligoprogression.
CI, confidence interval; HR, hazard ratio; OP‐OS, overall survival after oligoprogression; OS, overall survival.

**Table S1.** Details of the driver mutations and other nondriver gene alterations detected in patients analyzed using next‐generation sequencing.
**Table S2**. Univariate and multivariate analyses of factors associated with OP‐OS.
**Table S3**. Characteristics of patients who underwent ribonucleic sequencing (RNA‐seq).

## Data Availability

The data that support the findings of this study are available from the corresponding author upon reasonable request.
