# Supplementary material for: Frequency and Prognostic Impact of Local Ablation Therapy for Oligoprogression in Non‐Small Cell Lung Cancer
Source: Thorac Cancer. 2025 Jul 8;16(13):e70119. doi: 10.1111/1759-7714.70119 (PMC12238320; doi:10.1111/1759-7714.70119)
Supplement: Supplementary file 1 — Figure S1. Kaplan–Meier curve and estimated median progression‐free survival (A), local progression‐free survival (B), overall survival (C), and overall survival after oligoprogression (D) by treatment modality in patients with driver mutations. CI, confidence interval; HR, hazard ratio; LAT, local ablation therapy; LPFS, local progression‐free survival; OP, oligoprogression; OP‐OS, overall survival after oligoprogression; OS, overall survival; PFS, progression‐free survival. [file TCA-16-e70119-s002.pptx]

## Slide 1
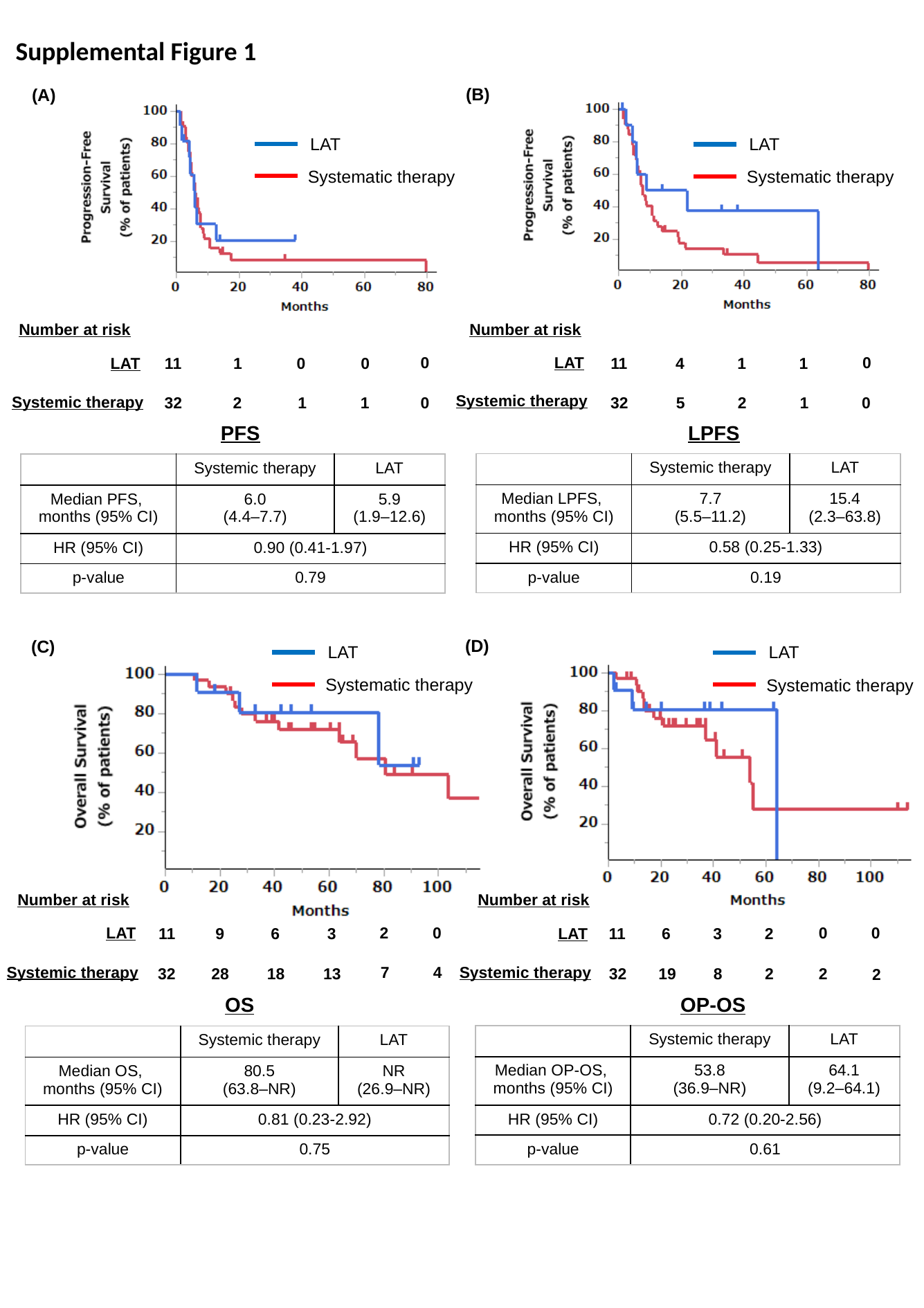

Supplemental Figure 1
(B)
(A)
LAT
LAT
Systematic therapy
Systematic therapy
Number at risk
Number at risk
0
0
LAT
LAT
0
0
1
1
11
1
11
4
Systemic therapy
Systemic therapy
32
0
32
0
2
1
1
5
2
1
PFS
LPFS
| | Systemic therapy | LAT |
| --- | --- | --- |
| Median LPFS, months (95% CI) | 7.7 (5.5–11.2) | 15.4 (2.3–63.8) |
| HR (95% CI) | 0.58 (0.25-1.33) | |
| p-value | 0.19 | |
| | Systemic therapy | LAT |
| --- | --- | --- |
| Median PFS, months (95% CI) | 6.0 (4.4–7.7) | 5.9 (1.9–12.6) |
| HR (95% CI) | 0.90 (0.41-1.97) | |
| p-value | 0.79 | |
(D)
(C)
LAT
LAT
Systematic therapy
Systematic therapy
Number at risk
Number at risk
0
2
0
0
LAT
9
6
11
LAT
3
2
3
11
6
Systemic therapy
Systemic therapy
4
7
13
18
32
28
2
8
2
32
19
2
OS
OP-OS
| | Systemic therapy | LAT |
| --- | --- | --- |
| Median OP-OS, months (95% CI) | 53.8 (36.9–NR) | 64.1 (9.2–64.1) |
| HR (95% CI) | 0.72 (0.20-2.56) | |
| p-value | 0.61 | |
| | Systemic therapy | LAT |
| --- | --- | --- |
| Median OS, months (95% CI) | 80.5 (63.8–NR) | NR (26.9–NR) |
| HR (95% CI) | 0.81 (0.23-2.92) | |
| p-value | 0.75 | |
