# Supplementary material for: Frequency and Prognostic Impact of Local Ablation Therapy for Oligoprogression in Non‐Small Cell Lung Cancer
Source: Thorac Cancer. 2025 Jul 8;16(13):e70119. doi: 10.1111/1759-7714.70119 (PMC12238320; doi:10.1111/1759-7714.70119)
Supplement: Supplementary file 2 — Figure S2. Kaplan–Meier curve and estimated median progression‐free survival (A), local progression‐free survival (B), overall survival (C), and overall survival after oligoprogression (D) by treatment modality in patients without driver mutations. CI, confidence interval; HR, hazard ratio; LAT, local ablation therapy; LPFS, local progression‐free survival; OP, oligoprogression; OP‐OS, overall survival after oligoprogression; OS, overall survival; PFS, progression‐free survival. [file TCA-16-e70119-s001.pptx]

## Slide 1
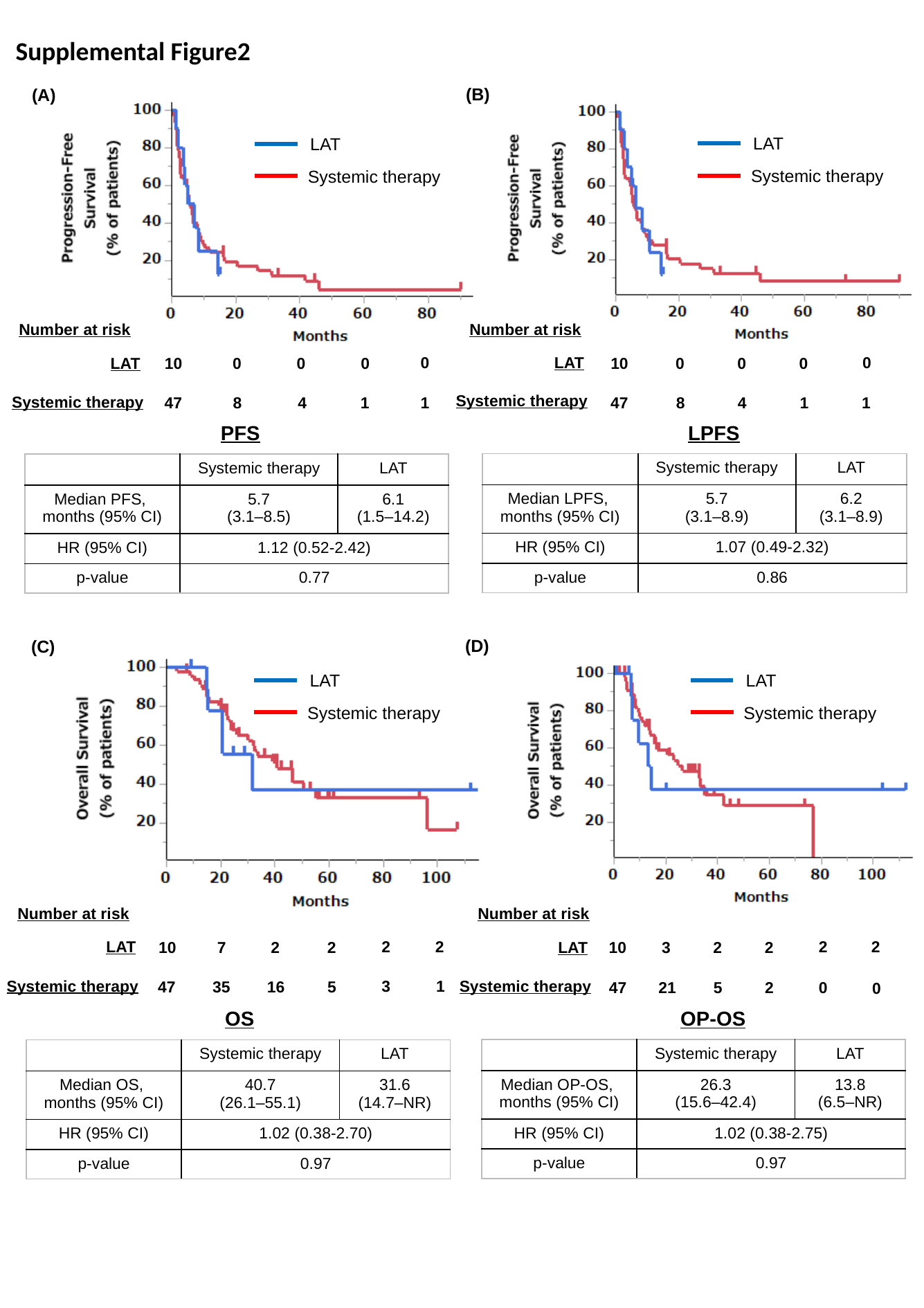

Supplemental Figure2
(B)
(A)
LAT
LAT
Systemic therapy
Systemic therapy
Number at risk
Number at risk
0
0
LAT
LAT
0
0
0
0
10
0
10
0
Systemic therapy
Systemic therapy
47
1
47
1
8
4
1
8
4
1
PFS
LPFS
| | Systemic therapy | LAT |
| --- | --- | --- |
| Median LPFS, months (95% CI) | 5.7 (3.1–8.9) | 6.2 (3.1–8.9) |
| HR (95% CI) | 1.07 (0.49-2.32) | |
| p-value | 0.86 | |
| | Systemic therapy | LAT |
| --- | --- | --- |
| Median PFS, months (95% CI) | 5.7 (3.1–8.5) | 6.1 (1.5–14.2) |
| HR (95% CI) | 1.12 (0.52-2.42) | |
| p-value | 0.77 | |
(D)
(C)
LAT
LAT
Systemic therapy
Systemic therapy
Number at risk
Number at risk
2
2
2
2
LAT
7
2
10
LAT
2
2
2
10
3
Systemic therapy
Systemic therapy
1
3
5
16
47
35
2
5
0
47
21
0
OS
OP-OS
| | Systemic therapy | LAT |
| --- | --- | --- |
| Median OP-OS, months (95% CI) | 26.3 (15.6–42.4) | 13.8 (6.5–NR) |
| HR (95% CI) | 1.02 (0.38-2.75) | |
| p-value | 0.97 | |
| | Systemic therapy | LAT |
| --- | --- | --- |
| Median OS, months (95% CI) | 40.7 (26.1–55.1) | 31.6 (14.7–NR) |
| HR (95% CI) | 1.02 (0.38-2.70) | |
| p-value | 0.97 | |
