# Supplementary material for: Frequency and Prognostic Impact of Local Ablation Therapy for Oligoprogression in Non‐Small Cell Lung Cancer
Source: Thorac Cancer. 2025 Jul 8;16(13):e70119. doi: 10.1111/1759-7714.70119 (PMC12238320; doi:10.1111/1759-7714.70119)
Supplement: Supplementary file 3 — Figure S3. Patient flowchart after treatment for oligoprogression. OP, oligoprogression. [file TCA-16-e70119-s003.pptx]

## Slide 1
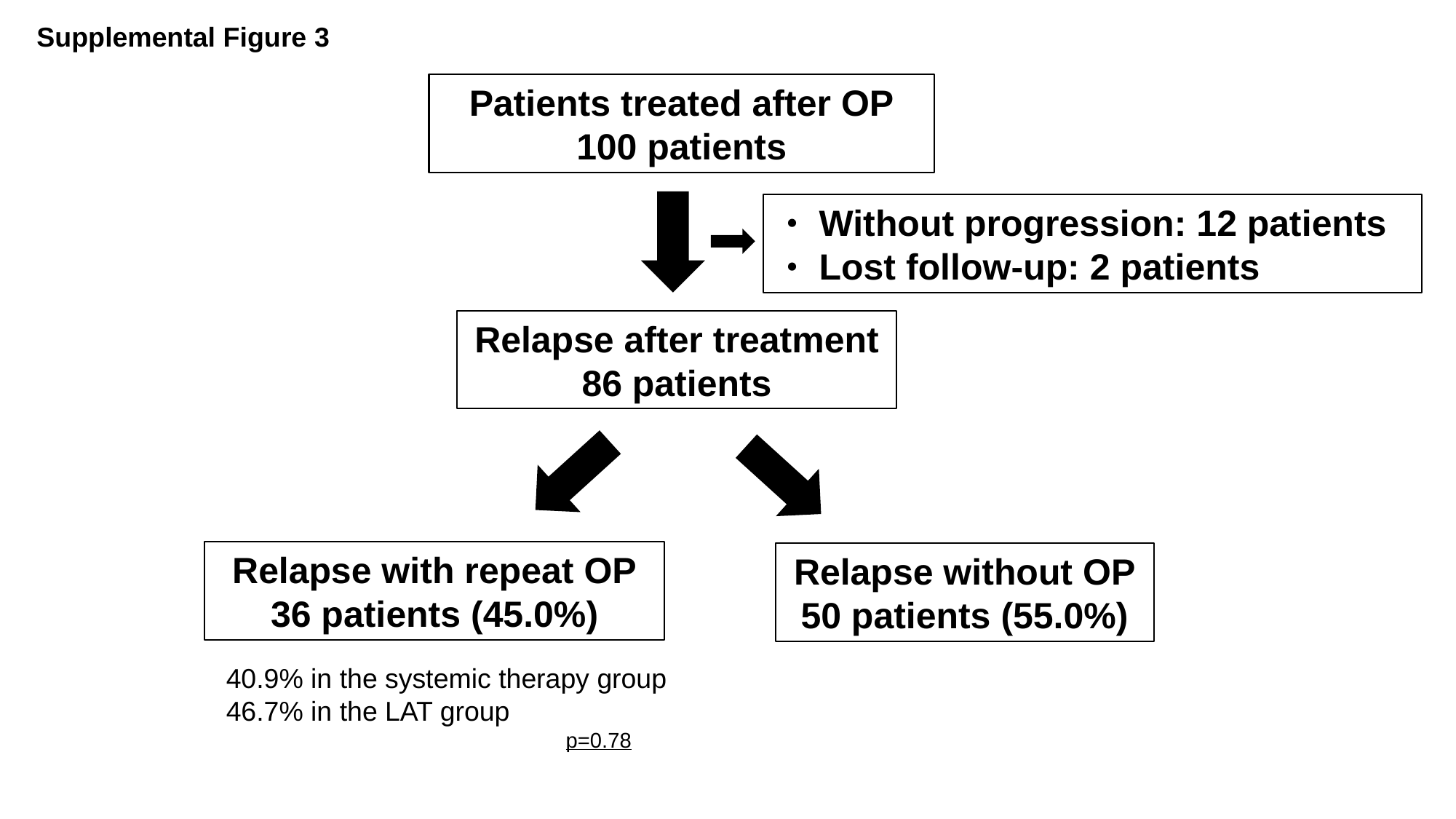

Supplemental Figure 3
Patients treated after OP
100 patients
・Without progression: 12 patients
・Lost follow-up: 2 patients
Relapse after treatment
86 patients
Relapse with repeat OP
36 patients (45.0%)
Relapse without OP
50 patients (55.0%)
40.9% in the systemic therapy group
46.7% in the LAT group
p=0.78
