# Supplementary material for: Frequency and Prognostic Impact of Local Ablation Therapy for Oligoprogression in Non‐Small Cell Lung Cancer
Source: Thorac Cancer. 2025 Jul 8;16(13):e70119. doi: 10.1111/1759-7714.70119 (PMC12238320; doi:10.1111/1759-7714.70119)
Supplement: Supplementary file 4 — Figure S4. Kaplan–Meier curve and estimated median overall survival after oligoprogression of patients with repeat oligoprogression. CI, confidence interval; HR, hazard ratio; OP‐OS, overall survival after oligoprogression; OS, overall survival. [file TCA-16-e70119-s004.pptx]

## Slide 1
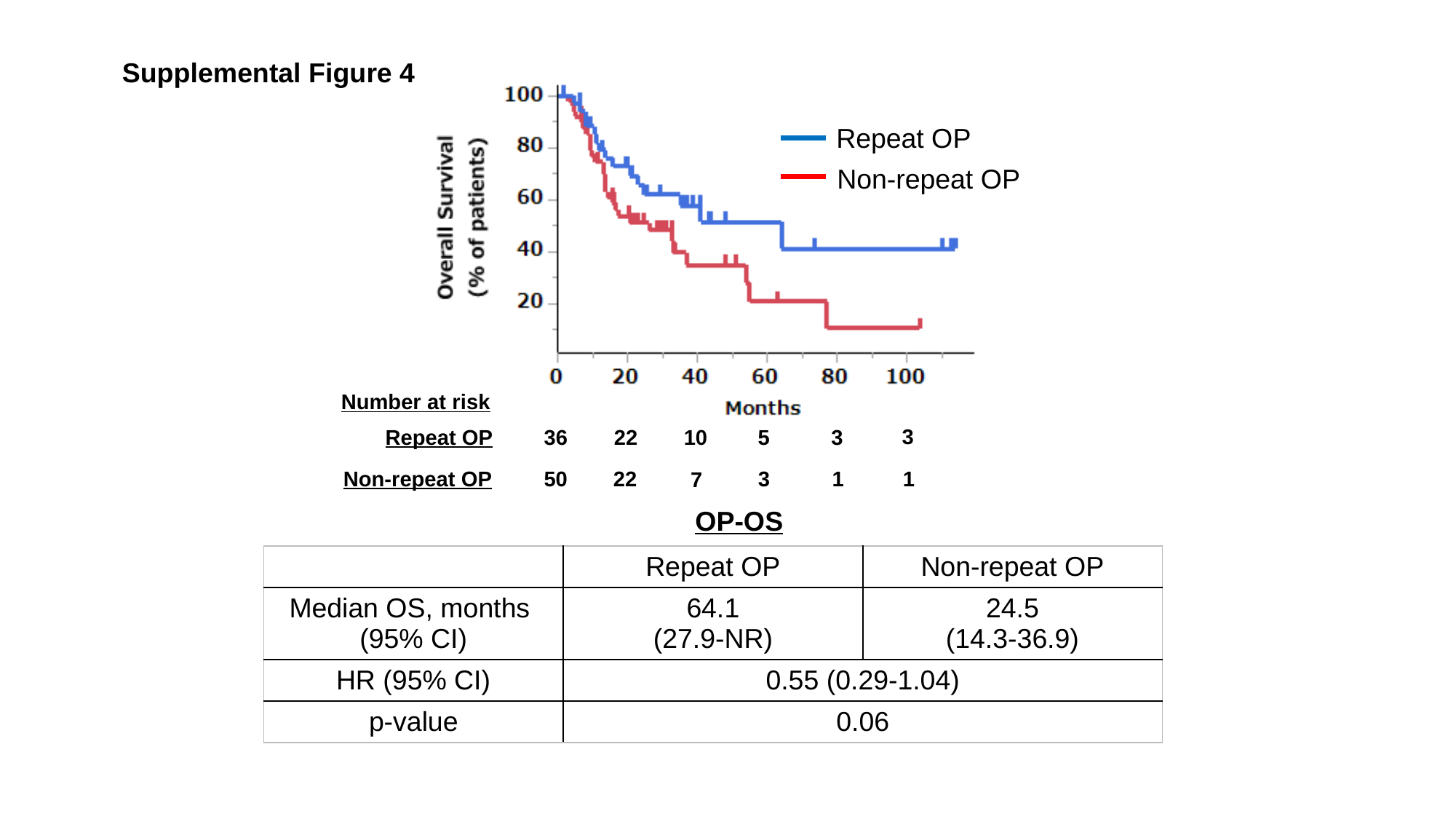

Supplemental Figure 4
Repeat OP
Non-repeat OP
Number at risk
3
5
Repeat OP
10
3
36
22
Non-repeat OP
50
1
22
3
1
7
OP-OS
| | Repeat OP | Non-repeat OP |
| --- | --- | --- |
| Median OS, months (95% CI) | 64.1 (27.9-NR) | 24.5 (14.3-36.9) |
| HR (95% CI) | 0.55 (0.29-1.04) | |
| p-value | 0.06 | |
