# Supplementary material for: Frequency and Prognostic Impact of Local Ablation Therapy for Oligoprogression in Non‐Small Cell Lung Cancer
Source: Thorac Cancer. 2025 Jul 8;16(13):e70119. doi: 10.1111/1759-7714.70119 (PMC12238320; doi:10.1111/1759-7714.70119)
Supplement: Supplementary file 5 — Table S1. Details of the driver mutations and other nondriver gene alterations detected in patients analyzed using next‐generation sequencing. Table S2. Univariate and multivariate analyses of factors associated with OP‐OS. Table S3. Characteristics of patients who underwent ribonucleic sequencing (RNA‐seq). [file TCA-16-e70119-s005.docx]

**Table S1:** Details of the driver mutations and other non-driver gene alterations detected in patients analyzed using next-generation sequencing.

**Driver mutations**

|  | Systemic treatment group  (n = 31) | LAT group  (n = 11) |
| --- | --- | --- |
| *EGFR* | 24 | 8 |
| *Exon21 L858R* | 12 | 5 |
| *Exon19 deletion* | 11 | 2 |
| *others* | 1 | 1 |
| *ALK* | 4 | 0 |
| *KRAS* G12C | 0 | 1 |
| *MET* Exon14  Skipping | 2 | 2 |
| *RET* | 1 | 0 |

**Other gene mutations**

|  | Systemic treatment group  (n = 31) | LAT group  (n = 11) |
| --- | --- | --- |
| *PIK3CA* | 2 | 3 |
| *TP53* | 3 | 0 |
| *CTNNB1* | 2 | 0 |
| *FGFR1* amp | 1 | 1 |
| *FGFR3* amp | 2 | 0 |
| *KRAS* G12V | 2 | 0 |
| *BRAF* G469R | 1 | 0 |
| *ERBB2* amp | 1 | 0 |
| *HMBS* | 1 | 0 |
| *LPR1* | 1 | 0 |
| *MYC* | 1 | 0 |

**Table S2.** Univariate and multivariate analyses of factors associated with OP-OS.

|  |  | **Univariate** | | | **Multivariate** | | |
| --- | --- | --- | --- | --- | --- | --- | --- |
| **Parameter** | **Category** | **HR** | **95% CI** | ***p*-value^a^** | **HR** | **95% CI** | ***p*-value^a^** |
| Age at OP | ≥75 years (vs. <75 years) | 1.62 | 0.83–3.15 | 0.16 |  |  |  |
| Sex | Female (vs. male) | 0.72 | 0.40–1.30 | 0.28 |  |  |  |
| Treatment line | 2nd (vs. 3^rd^ and after) | 1.96 | 0.94–4.07 | 0.07 |  |  |  |
| Driver mutation | With (vs. without) | 0.49 | 0.27–0.92 | 0.03 | 0.44 | 0.23–0.85 | 0.01 |
| PD-L1 expression | ≥50% (<50%) | 1.06 | 0.45–2.48 | 0.90 |  |  |  |
| LAT | With (vs. without) | 0.79 | 0.36–1.71 | 0.55 |  |  |  |
| Disease progression only in intrathoracic lesions | Yes (vs. No) | 1.72 | 0.87–3.42 | 0.12 |  |  |  |
| Disease progression only in liver lesions | Yes (vs. No) | 4.70 | 1.12–19.7 | 0.03 | 3.83 | 0.81–18.1 | 0.09 |
| Disease progression only in brain lesions | Yes (vs. No) | 0.45 | 0.11–1.85 | 0.27 |  |  |  |
| Disease progression only in bone lesions | Yes (vs. No) | 1.77 | 0.54–5.80 | 0.35 |  |  |  |
| Number of lesions | 1 (vs. 2–3) | 0.93 | 0.51–1.71 | 0.82 |  |  |  |
| Number of organs atdisease progression | 1 (vs. 2–3) | 1.00 | 0.35–2.80 | 0.99 |  |  |  |
| PFS of previous treatment | ≥6 months (<6 months) | 0.32 | 0.17–0.59 | <0.01 | 0.45 | 0.18–0.65 | <0.01 |
| Repeat OP | Yes (vs. No) | 0.55 | 0.29–1.04 | 0.07 |  |  |  |

Hazard ratios (HRs) and 95% confidence intervals (CIs) were estimated using the Cox proportional hazards regression model.

^a^p < 0.05 was considered statistically significant.

Abbreviations: LAT, local ablation therapy; OP, oligoprogression; OS, overall survival; PFS, progression-free survival.

**Table S3.** Characteristics of patients who underwent RNA sequencing (RNA-seq).

**Pre-oligoprogression tissues**

| Pt | Sex | Histology | Driver mutation | Lesion site |
| --- | --- | --- | --- | --- |
| 1^※^ | F | Ad | None | Lung |
| 2^※^ | F | Ad | EGFR L858R | Lung |
| 3 | F | Ad | None | Brain |

**Post-oligoprogression tisses**

| Pt | Sex | Histology | Driver mutation | Treatment line | Lesion site | Modality | PFS (M) |
| --- | --- | --- | --- | --- | --- | --- | --- |
| 1^※^ | F | Ad | None | 2nd | Lung | Surgery | 23.2 |
| 2^※^ | F | Ad | EGFR L858R | 2nd | Lung | Surgery + systemic therapy | 24.7 |
| 4 | M | NOS | None | 5th | Lung | Surgery + systemic therapy | 5.7 |
| 5 | M | Ad | EGFR exon19del | 7th | Lung | Surgery | 4.3 |
| 6 | F | Ad | ALK | 5th | Lung, Brain | RT+ surgery | 7.8 |
| 7 | M | Ad | None | 3rd | Lung | Systemic therapy | 11.8 |
| 8 | F | Sq | None | 2nd | Lung | Systemic therapy | 1.1 |
| 9 | F | Ad | KRAS G12C | 2nd | Lung | Surgery | 2.3 |

Asterisks (*) indicate patients for whom samples were obtained at diagnosis and after OP.
